# Blue light irradiation and its beneficial effect on Dupuytren’s fibroblasts

**DOI:** 10.1371/journal.pone.0209833

**Published:** 2019-01-11

**Authors:** Julia Krassovka, Annika Borgschulze, Benita Sahlender, Tim Lögters, Joachim Windolf, Vera Grotheer

**Affiliations:** Department of Trauma and Hand Surgery, Medical Faculty, Heinrich-Heine-University Düsseldorf, Düsseldorf, Germany; Massachusetts General Hospital, UNITED STATES

## Abstract

Dupuytren’s contracture is a fibroproliferative disorder affecting the palmar fascia of the hand. Most affected are the ring fingers, and little fingers of middle-aged men. Symptomatic for this disease is the increased proliferation and differentiation of fibroblasts to myofibroblasts, which is accompanied by an elevated α-SMA expression. The present study evaluated the therapeutic benefit of blue light (λ = 453 nm, 38 mW/cm^2^, continuous radiance, spot size 10–12 cm^2^) as well as the molecular mechanism mediating this effect. It could be determined that blue light significantly diminished the induced α-SMA protein expression in both normal palmar fibroblasts and Duypuytren’s fibroblasts. The beneficial effect mediated by this irradiance, radiant exposure and wavelength was associated with an elevated reactive oxygen species generation. Furthermore, the data underlines the potential usefulness of blue light irradiation as a promising therapy option for Dupuytren’s disease, especially for relapse prevention, and may represent a useful strategy to treat further fibrotic diseases, such as keloids, hypertrophic scarring, and scleroderma.

## Introduction

The cause of Dupuytren’s disease (DD) is believed to be multifactorial with a genetic disposition and concerns predominantly middle-aged Caucasian men [1]. It’s a benign fibromatosis affecting the palmar fascia of the hand, characterized by an increased subcutaneous nodule, followed by cord formation dependent on the progression of this disease. Responsible for cord or nodule formation is an excessive proliferation and differentiation of fibroblasts to myofibroblasts, so that the myofibroblasts becomes the predominant cell type in Dupuytren’s tissue [2]. And the benign but undesired growth of Dupuytren’s myofibroblasts is supported by their decreased susceptibility to undergo apoptosis [3–5]. In general, differentiated myofibroblasts show characteristics of fibroblasts and smooth muscle cells, and express α-smooth muscle actin (α-SMA), which allows the cells to contract. Therefore myofibroblasts assume mandatory functions in wound healing enabling wound closure. Hence, the common method to identify (Dupuytren’s) myofibroblasts is proving the intracellular α-SMA-amount. The pathological staging of DD is based on the definition given by Luck et al. [6], who suggested to differentiate between proliferative, involutional, and residual stages. In the proliferative phase, α-SMA-expression increases due to the uncontrolled proliferation of myofibroblasts, leading to nodule formation influenced by local mediators such as transforming growth factor-β1 (TGF-β1). During the progression of Dupuytren’s disease myofibroblasts synthesize an elevated level of α-SMA, collagen III and fibronectin [7]. So, the involutional stage is marked by an elevated proportion of collagen III, resulting in the formation of the characteristic cords and an increased extracellular matrix deposition. In the residual phase, nodules and myofibroblasts decrease and α-SMA and collagen III were diminished [8], and replaced by scar tissue.

Furthermore, TGF-β1 seems to play a superordinate role in the formation and maintenance of DD, especially because TGF-β1 induces the expression of α-SMA, collagen III and fibronectin, and has been shown to increase the contraction of myofibroblasts [9, 10]. So, TGF-β is localized in myofibroblasts in all phases of DD [1]. This is not surprising, since TGF-β1 is an abundant pro-inflammatory cytokine, involved in pathological scarring and fibrosis [11].

One therapy option for advanced DD is the partial fasciectomy, which is unfortunately accompanied by significant recurrence rates [12]. This surgical technique removes involved tissue, and contains extensive dissection of diseased longitudinal cords or nodules, which are removed from the surrounding fascia [13]. Needle fasciotomy or enzyme injection as collagenase [14] were performed as alternative methods, and at least 88% of the collagenase-treated patients were observed to have their movement improved by up to 48° movement in a one-year follow-up study [15].

The use of photobiomodulation-based therapies as a treatment option for DD is a promising alternative, which has not been applied to this day. Visible light could mediate its therapeutic effect via the interaction with endogenous photoreceptors, chromophores such as porphyrins, cytochrome c oxidase, nitrosated and flavo- proteins, opsins, and ion-gate channels [16, 17], dependent on the specific characteristics of the irradiated tissue.

It has already been shown, that blue light, dependent on the wavelength, intensity (irradiance) and radiant exposure, has anti-inflammatory effects in keratinocytes [18] and suppresses dendritic cell activation [19]. In rodents blue light inhibits skin tumors [20] and improves wound healing [21]. But, in the context of DD, blue light is particularly interesting, because mitosis and proliferation of cultured cells could be inhibited [22]. Furthermore, the proliferation and induced α-SMA-expression in human dermal myofibroblasts could be diminished [23]. These preconditions could render the irradiance with blue light (λ = 453 nm, intensity 38 mW/cm^2^) an interesting tool for the treatment of Dupuytren’s fibroblasts. Therefore, the therapeutic benefit of blue light (with these parameters) on Dupuytren’s disease was evaluated in the present work.

## Material and methods

### Patients

Study approval was obtained from the Ethics Review Board of the Medical Faculty, Heinrich-Heine-University Düsseldorf (Study No. 3634). Of the 23 patients with Dupuytren’s disease (DD), 3 were female and 20 were male (average age 62 years), all of whom underwent fasciectomy at the Department of Trauma and Hand Surgery, University Hospital, Düsseldorf, Germany. Patients who suffered from carpal tunnel syndrome served as normal palmar fascia control (NPF). Their tissue was dissected from the palmar fascia and served as control group, if it had to be removed anyway (n = 24; 16 women and 8 men; average age 52.5 years). The usage of human material was in compliance with the Declaration of Helsinki Priniciples. Informed written consent was gathered from all patients. During surgery, tissue specimen were deposited in PBS (1% penicillin/streptomycin) and cooled to 4°C. Samples were processed within 24 h. All data were anonymized prior to analysis.

### Isolation and culture of the cells

All chemicals were obtained from Sigma-Aldrich, and cell culture materials from Cellstar (Greiner bio-one), if not mentioned otherwise.

Minced tissue was incubated at 37°C in 10 ml collagenase-solution (0.1 M CaCl, 0.005 M Glucose, 0.1 M Hepes, 0.12 M NaCl, 0.05 M KCl + 1.5% BSA and 0.2% collagenase type 1) for 45 min, filtrated through a 100 nm nylon strainer, centrifuged (1200 rpm, 5 min at 4°C) and the pellet resuspended in 30 ml 0.9% NaCl-solution. After centrifugation (5 min at 1200 rpm at 4°C) the pellet was resuspended in standard medium (RPMI 1640 Medium Biochrom with 10% FBS, 1 x NEAA, 1% penicillin/streptomycin, 1 x sodium pyruvate) and incubated at 37°C, 5% CO_2_.

### Irradiation and TGF-β1 treatment

Fibroblasts and NPF were used in passage 3 – 8. Cells were seeded in 3 × 10^4^/6-Well and irradiation was started after 24 h, so that procedure was started under subconfluent conditions. Fibroblasts and NPF were either treated with 2 ng/ml TGF-β1 (ImmunoTools rh TGF-β1), irradiated (0–80 Joule/cm^2^), treated and irradiated, or neither of those. The irradiation was performed in PBS. The appropriate control cells were put beside the irradiation unit and incubated with PBS equally for the same time. After that, PBS was replaced by culture medium. Temperature was checked with a thermometer, and the achieved maximum temperature was 37°C. Cells were harvested by cell-scraping and the α-SMA protein expression was measured with Western Blot Analysis as described down below. No technical repeats could be performed due to limited biological tissue samples.

### Blue light irradiation

All irradiation experiments were accomplished with a prototype of a narrow-band light-emitting diode (LED), which emits light of the wavelength λ = 453 nm (16.7 min). The power density was 38 mW/cm^2^. The cells were continuously irradiated from above in PBS, in 6-Well-Plates. 60 (6 × 10) LEDs were equally distributed over a total of 10–12 cm^2^. The blue light-emitting device used in our experiments was provided by Philips GmbH, Innovative Technologies, Aachen, Germany.

### Cell viability test (metabolic activity)

Fibroblasts were irradiated with different doses (5, 10, 20, 40, 60, 80 Joule/cm^2^). Then, effects were analyzed with CellTiter-Blue Assay (Promega, Madison, USA/G3582). CellTiter-Blue uses an indicator dye to measure the metabolic capacity of cells, as an indirect evidence for cell viability. CellTiter-Blue was added at a ratio of 1:20 into the medium. After an incubation time of 1 h, the fluorescence (540 _EX_/590_EM_) was measured in a 1420 Multilabel Counter (Victor^3^, PerkinElmer).

### Western blot analysis

In order to verify the differentiation from fibroblasts to myofibroblasts, the α-SMA protein expression was measured with Western Blot analysis. Protein concentration was determined with the Pierce BCA Protein Assay Kit in line with the manufacturer’s instructions. 10 μg protein were mixed with the Laemmli buffer (4 × Tris-glycin-SDS sample buffer, 252 mmol TrisHCL pH 6.8; 40% Glycerin; 8% SDS; 0.01% bromphenol blue + 20% mercaptoethanol), denaturated for 5 min at 95°C, and separated on a 12% sodium dodecyl sulphate-polyacrylamide gel (SDS-PAGE)). Separated proteins were transferred to a nitrocellulose membrane (25 V– 1.0 A– 30 min) in a trans-blot system (BioRad Trans-Blot Turbo). Successful transfer was verified by Ponceau red S staining. The membrane was saturated with 5% BSA in TBST for 1 h (RT) and immunolabeled with mouse anti-human-α–SMA (Abcam/ab7817) (1:1000 in 3% BSA in TBST) over night at 4°C and mouse anti-human Glyerinaldehyd-3-phosphat-Dehydrogenase (GAPDH) antibody (BioRad/#12004167) (1:6000 in 3% BSA in TBST) for 1 h (RT). After washing with TBST, the membrane was incubated with horseradish peroxidase (HRP) conjugated goat anti-mouse secondary antibody (Dako/P0447) for 1 h (RT). Bound antibodies were detected using Clarity ^TM^ Western ECL Substrate (BioRad/#170–5060) and analyzed with the Quantity One 1-D Analysis Software Version 4.6.5 (BioRad).

### Reactive oxygen species (ROS) detection

24 h after seeding 3 × 10^4^ cells/6-Well, ROS detection was performed using Dihydrorhodamine 123 (DHR123; Sigma-Aldrich/D1054). DHR123 is oxidized to its fluorescent derivative Rhodamine 123 by unspecific ROS. In order to trigger a strong reaction, the irradiation set up was first conducted with 60 Joule/cm^2^, and then fibroblasts were incubated for a minimum of 30 min in DHR123 (10 μM) in standard medium. Cells were washed with PBS, and fluorescence was measured (485_EX_/535_EM_) in the 1420 Multilabel Counter (Victor^3^, PerkinElmer).

### Measuring apoptosis

Fibroblasts and NPF were either treated with 2 ng/ml TGF-β1, irradiated (40 Joule/cm^2^), treated and irradiated, or neither of those over the course of 5 days. After that, fibroblasts were treated with staurosporine (STS) (Sigma-Aldrich/S5921) (0.075 μM) for 18 h. Cells were then harvested and centrifuged (1200 rpm for 5 min). The supernatant was removed, and the pellet resuspended in 500 μl FACS buffer (cell wash + 3% FBS). Cells were set on ice, fixed with 70% EtOH for 20 min and centrifuged (1200 rpm for 5 min). Pellets were washed two times with FACS buffer and stained with 200 μl propidium iodide solution (20 μg/ml TBS-T; Sigma-Aldrich/P4170). Cells were incubated protected from light for 30 min (RT) and measured with FACS Calibur (BD Biosciences).

### Statistical analysis

Statistical analysis was performed with two-tailed *t*-test or two-way ANOVA and Mann–Whitney-U test, respectively. The data were expressed as mean value and standard deviation (SD). The level of significance was considered to be p ≤ 0.05.

## Results

### Cell viability (metabolic activity)

**Fig 1:**
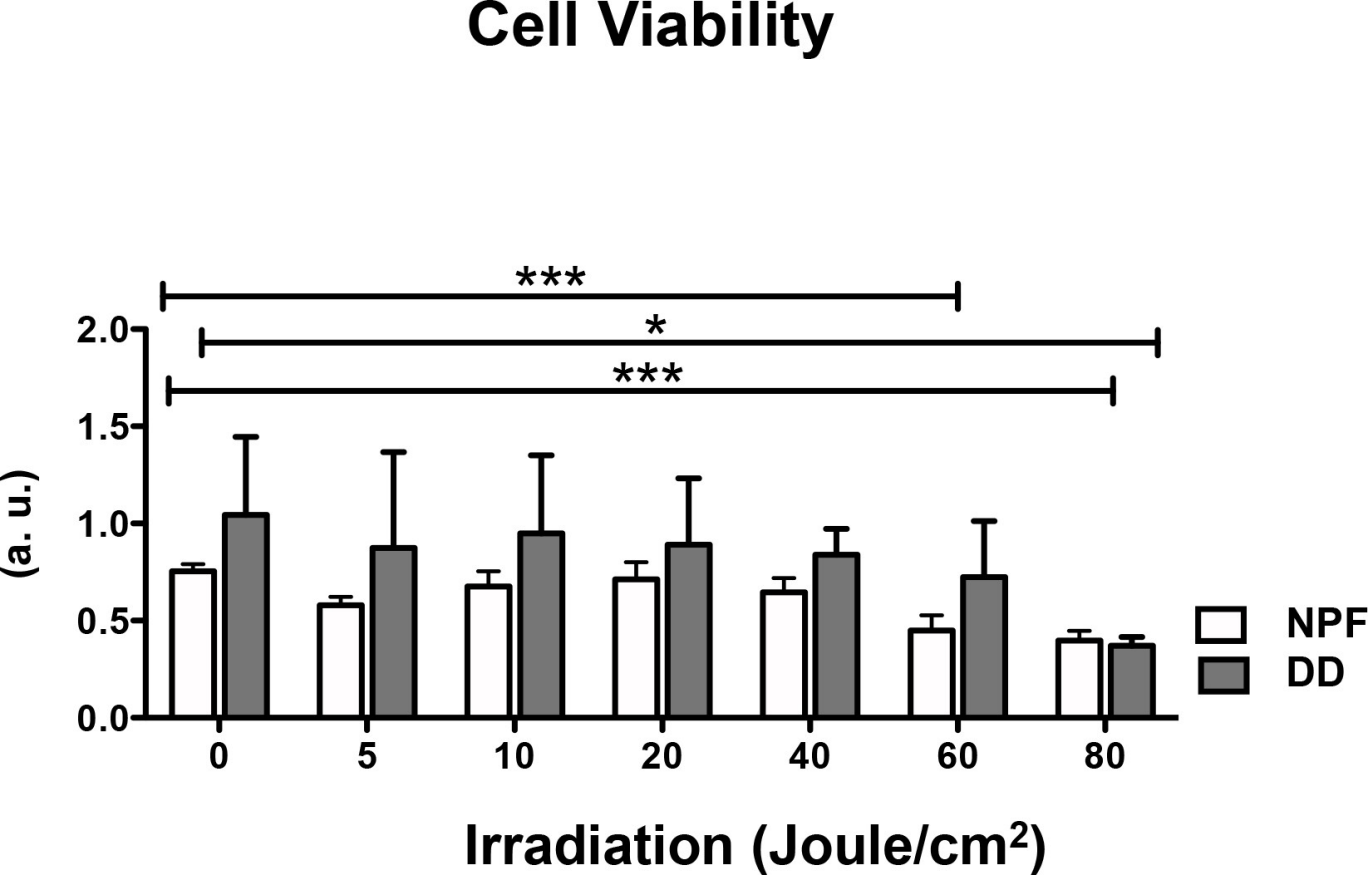
Cell viability (metabolic activity) after 24 h is shown. Fibroblasts of DD (n = 3–6) and NPF (n = 5) were irradiated with different doses (5, 10, 20, 40, 60, 80 Joule/cm^2^). * p ≤ 0.05; *** p ≤ 0.0005. Bars represent mean ± SD of individual experiments indicated.

As an indicator for cell viability, metabolic activity was analyzed (CellTiter-Blue Assay). From the beginning metabolic activity of DD fibroblasts was higher compared to all NPF (normal palmar fascia control) fibroblasts (Fig 1). From 60 Joule/cm^2^ on, metabolic activity was significantly reduced in NPF fibroblasts and in DD fibroblasts metabolic activity was notably reduced. From 80 Joule/cm^2^ on DD fibroblasts activity was significantly down regulated and in NPF fibroblasts highly significantly degraded, compared to their appropriated untreated controls (0 Joule/cm^2^) (Fig 1). Because of this a radiant exposure of 40 Joule/cm^2^ was used in our subsequent experiments.

### Irradiation-based modulation of α-SMA protein expression

**Fig 2:**
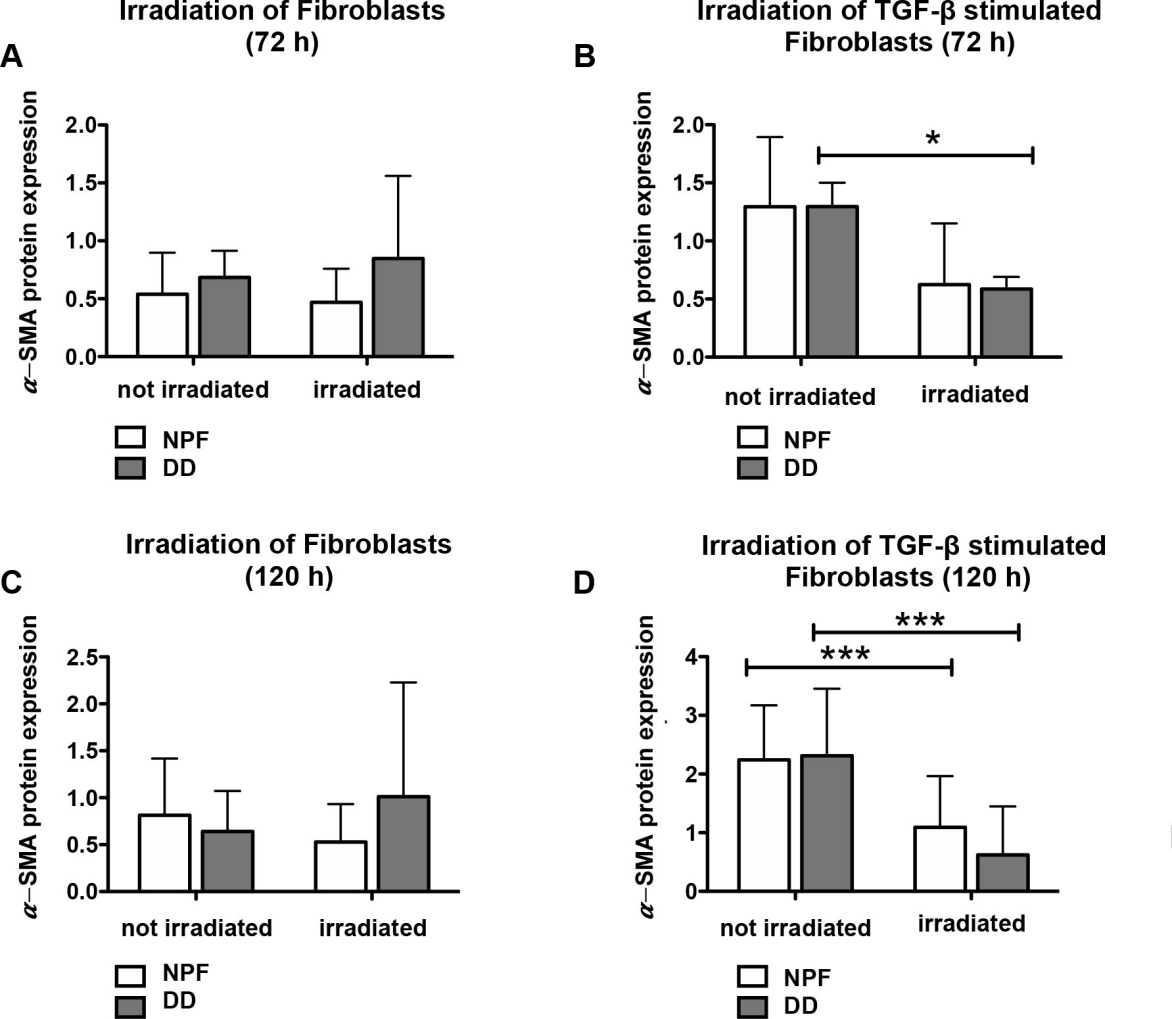
Relative α-SMA protein expression of resting or TGF-β1 stimulated and irradiated (40 Joule/cm^2^) NPF compared to DD fibroblasts. Irradiation had no significant effect on α*-*SMA protein expression of resting DD and NPF fibroblasts (Fig 2A and 2C). TGF-β1 treatment over 72 h and irradiation (40 Joule/cm^2^) declined α*-*SMA protein expression in DD fibroblasts (Fig 2B). The effect was slightly stronger after 120 h incubation (Fig 2D). **A** The α-SMA protein expression of resting and irradiated (72 h) fibroblasts of NPF (n = 8) and DD (n = 7) **B** The α-SMA protein expression of TGF-β1 stimulated and irradiated (72 h) NPF (n = 8) and DD (n = 7) **C** The α-SMA protein expression of resting and irradiated (120 h) NPF (n = 8) and DD (n = 6–7) **D** The α-SMA protein expression of TGF-β1 stimulated and irradiated (120 h) NPF (n = 8) and DD (n = 6–7). Protein expression was measured and normalized to the housekeeping gene GAPDH. * p ≤ 0.05; *** p ≤ 0.0005. Bars represent mean ±SD of individual experiments indicated.

**Fig 3:**
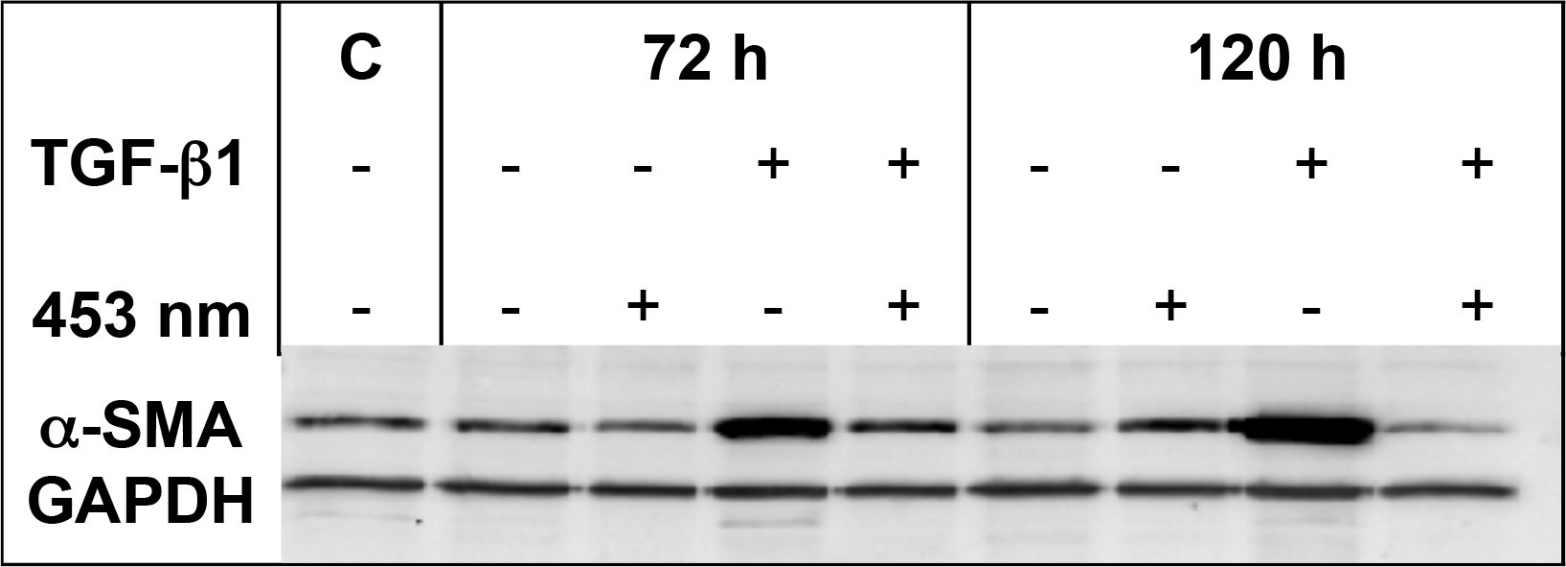
A representative western blot analysis showing α-SMA expression of DD fibroblasts with TGF-β1 stimulation (+), without stimulation (-), with (+) or without (-) irradiation at 453 nm (either about 72 or 120 h). The untreated DD control harvested after 24 h C α-SMA expression was measured and normalized to GAPDH.

In unstimulated DD and NPF fibroblasts blue light caused no significant effect on α*-*SMA protein expression (Fig 2A and 2C and Fig 3). TGF-β1 treatments over 72 h and blue light (40 Joule/cm2) decrease α*-*SMA protein expression in DD fibroblasts significantly (Fig 2B and Fig 3). The effect was even stronger after a 120 h incubation time (Fig 2D and Fig 3).

A blue light irradiation with the intensity of 38 mW/cm^2^ and with a radiant exposure of 40 Joule/cm^2^ with a narrow-band LED, which emits light of the wavelength λ = 453 nm down regulated α*-*SMA protein expression significantly in DD fibroblasts after TGF-β1-treatment. The α*-*SMA protein expression decreases the longer the treatment continues.

### ROS expression after irradiation

**Fig 4:**
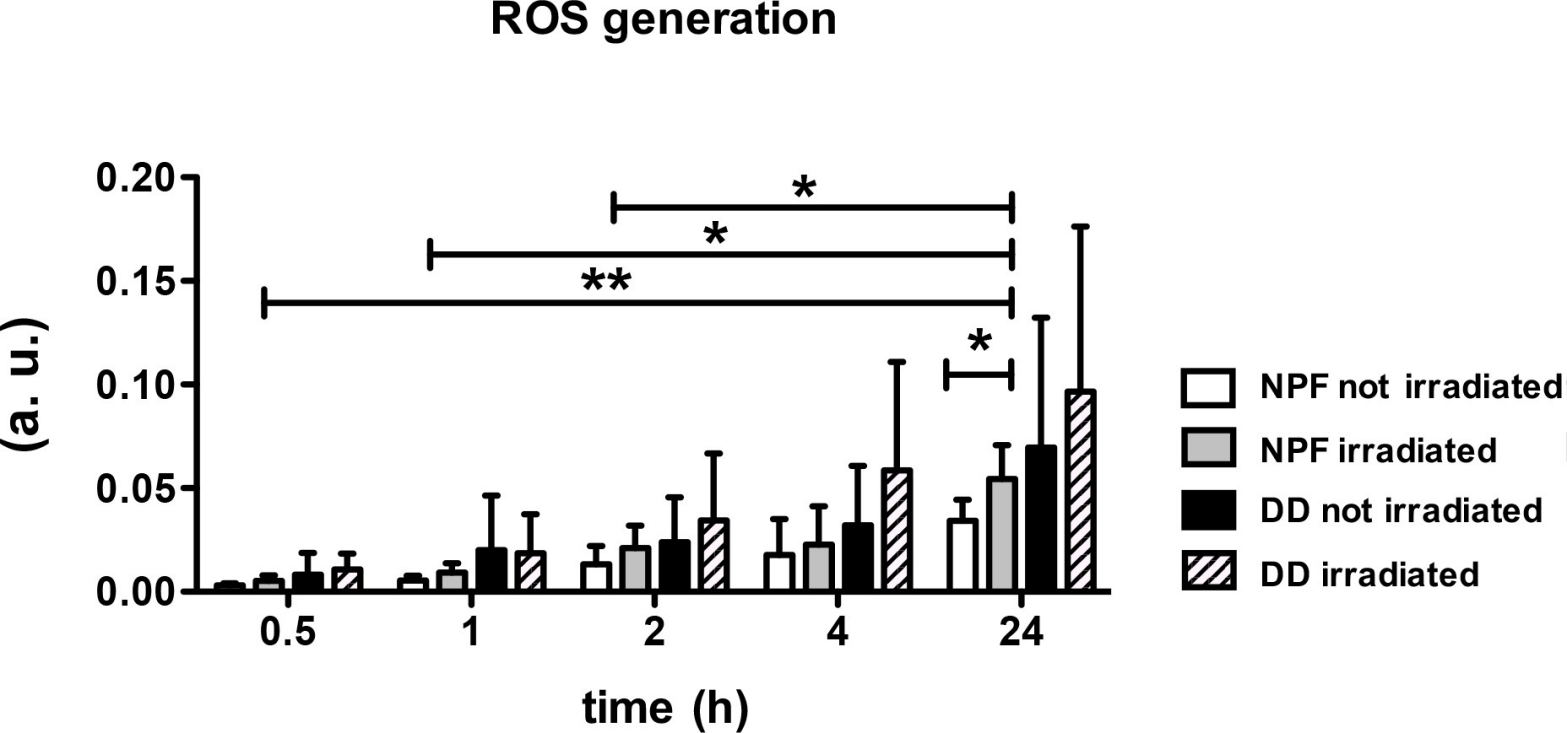
Analysis of ROS in DD compared to NFF. **A** ROS generation of irradiated (60 Joule/cm^2^) or resting NPF (n = 5) compared to DD fibroblasts (n = 5). Measurement after 0.5, 1, 2, 4, and 24 h with DHR123. Absolute ROS expression of fibroblasts increased after irradiation. Both irradiated and non-irradiated fibroblasts of DD always revealed the highest ROS expression compared to irradiated and non-irradiated NPF fibroblasts (Fig 4A). ROS expression rose over 24 h continuously. Nevertheless, the ROS expression of irradiated NPF fibroblasts was significantly enhanced after 24 h compared to irradiated NPF fibroblasts after 0.5, 1, and 2 h. The irradiated NPF fibroblasts showed a significant boost after 24 h compared to non-irradiated NPF fibroblasts.

From the beginning on DD fibroblasts have generated more ROS in tendency. Absolute ROS increased after irradiation in both fibroblasts’ groups NPF and DD. Control DD fibroblasts as well as irradiated ones generated more ROS compared to their appropriate NPF controls (Fig 4A). So, ROS expression measured with DHR123 rose continuously over 24 h. The ROS expression of irradiated NPF fibroblasts was significantly enhanced after 24 h, compared to irradiated NPF fibroblasts after 0.5, 1, and 2 h. After 24 h irradiated NPF fibroblasts showed a significantly higher amount of ROS in comparison to not irradiated NPF fibroblasts.

Furthermore, the use of blue light appears to elevate ROS -especially in Dupuytren´s fibroblasts-.

### The apoptotic potential of DD fibroblasts

In untreated DD and NPF fibroblasts apoptosis rate was similar. Same effect could be shown if fibroblasts were treated with STS, even if the rate of apoptosis was higher overall. If cells were treated daily with TGF-β1, the rate of apoptosis was significantly higher in DD fibroblasts, compared to NPF. Overall the results demonstrated that the treatment with blue light did not induce apoptosis. And the treatment with blue light and an additional application with TGF-β1 slightly reduced apoptosis in NPF and DD fibroblasts. So, in conclusion the therapeutic benefit of blue light is not regulated via apoptosis.

## Discussion

Partial fasciectomy, enzyme injection, and needle fasciotomy are todays treatment options for Dupuytren’s disease (DD) [24]. Due to the limitations of these treatment methods, researchers and clinicians are forced to search for better therapies. The usage of photobiomodulation strategies may provide a solution to this problem, because the combination of intensity, radiant exposure, wavelength, and their specific interaction with the absorption properties of the target tissue can have a therapeutic effect [25]. So, blue light (50 mW/cm^2^; 15, 30 Joule/cm^2^; λ = 420 nm) has already been proven to inhibit TGF-β-induced α-SMA expression in differentiated human dermal fibroblasts [23]. Especially in Dupuytren’s fibroblasts, the increased and pathological differentiation of fibroblasts to myofibroblasts, is associated with an elevated α-SMA-expression. Hence, in this study the therapeutic benefit of blue light irradiation on Dupuytren’s fibroblasts was analyzed for the first time.

Dupuytren’s fibroblasts and normal palmar fascia (NPF) fibroblasts were irradiated (with a wavelength of λ = 453 nm and an intensity of 38 W/cm^2^), and as the most promising radiant exposure 40 Joule/cm^2^ was used for the subsequent experimental procedures (Fig 1). Since the irradiation with 40 Joule/cm^2^ hardly inhibited the cell viability/metabolic activity of Dupuytren’s fibroblasts and especially of NPF, which is required for a therapeutic application of blue light in DD.

Up to 60 Joule/cm^2^ irradiation, metabolic activity of Dupuytren’s fibroblasts was increased compared to NPF (Fig 1). This result is supported by several research groups, demonstrating that the proliferation of Dupuytren’s fibroblasts is elevated compared to control cells by the activation of the TGF-β/SMAD- and extracellular signal-regulated kinase (ERK) signaling [26], and an autocrine regulation through epidermal growth factor (ERBB)-2 and insulin growth factor (IGF)-1 receptors, as well as Akt-signaling [27].

Furthermore, it could be demonstrated that after 24 h cell viability of Dupuytren’s fibroblasts appeared to be considerably higher than NPF fibroblasts viability (Fig 1, 0 Joule), although in both groups the same cell number was seeded. This is a remarkable result, because “cell viability” was indirectly determined by measuring metabolic activity (CellTiter-Blue Assay). These results and the elevated ROS amount in Dupuytren’s fibroblasts compared with NPF fibroblasts shown in Fig 4 indicate, that Dupuytren’s fibroblasts have, by nature, a higher metabolism than the respective controls from patients suffering from carpal tunnel syndrome. And this may be an explanation why DD fibroblasts were more affected by the blue light irradiation, than the NPF fibroblasts. This assumption is solidified by the findings, that the expression of the housekeeping gene Glyerinaldehyd-3-phosphat-Dehydrogenase (GAPDH) is increased in Dupuytren’s fibroblasts (data not shown) treated with TGF-β1 compared to NPF.

It could be ascertained that the exposure with λ = 453 nm does not change α-SMA expression in neither resting NPF nor in Dupuytren’s fibroblasts after 72 h and 120 h (Fig 2A and 2C and Fig 3). (It has to be considered, that only one donor causes those high standard deviations of irradiated Dupuytren’s fibroblasts.) Furthermore, to tighten standard deviation is difficult in Dupuytren’s research, because it is a multifactorial disease, and tissue specimen were not classified in regard to secondary diseases like diabetes, alcohol abuse, smoking, or just relapse. And to exacerbate these issues, the amount of tissue specimen (quantity of Dupuytren’s tissue specimen is low, but quantity of NPF tissue specimen is even lower) is not sufficient for technical replicates. But interestingly, if NPF were pretreated with TGF-β1 and irradiated with blue light, α-SMA protein expression was significantly diminished after 72 h, and this effect could be further enhanced after 120 h (Fig 2B and 2D and Fig 3). And special attention should be paid to the observation, that in TGF-β1 pretreated Dupuytren’s fibroblasts, α-SMA protein expression is significantly decreased by the blue light irradiation (Fig 2B and 2D and Fig 3).

It was assumed that the effect of the blue light irradiation is mediated by the generation of reactive oxygen species (ROS), such as singlet oxygen [28] or hydrogen peroxide (H_2_O_2_) [23]. And it was presumed, that photons interact with endogenous photoreceptor molecules, such as lipofuscin [29], cytochrome c oxidase [30], or flavin-based photo sensors [31]. In fact, Taflinski et al. determined that an irradiation with blue light (420 nm) led to an increased amount of ROS as well as α-SMA reduction, and the induction of intracellular oxidative stress was thought to be the cause for that [23]. These findings intrigued the present work to examine ROS in Dupuytren’s fibroblasts after the exposure to blue light. It could be assured, that Dupuytren’s fibroblasts tended to produce more ROS, which was detected (after 24 h seeding the same cell number) with DHR123, an unspecific ROS indicator (Fig 4). As mentioned before, this might be due to a higher metabolic activity of Duypuytren’s fibroblasts. And the irradiation in general induced increased ROS formation in DD and NPF fibroblasts in tendency (Fig 4). As postulated by Taflinski et al., we assume that repeated smaller doses of blue light irradiation induce subtoxic levels of intracellular oxidative stress (as shown here in Fig 4), which may induce energy-consuming cellular responses against oxidative stress, which may in turn result in a proliferation stop and interfere with myofibroblasts differentiation [23].

**Fig 5:**
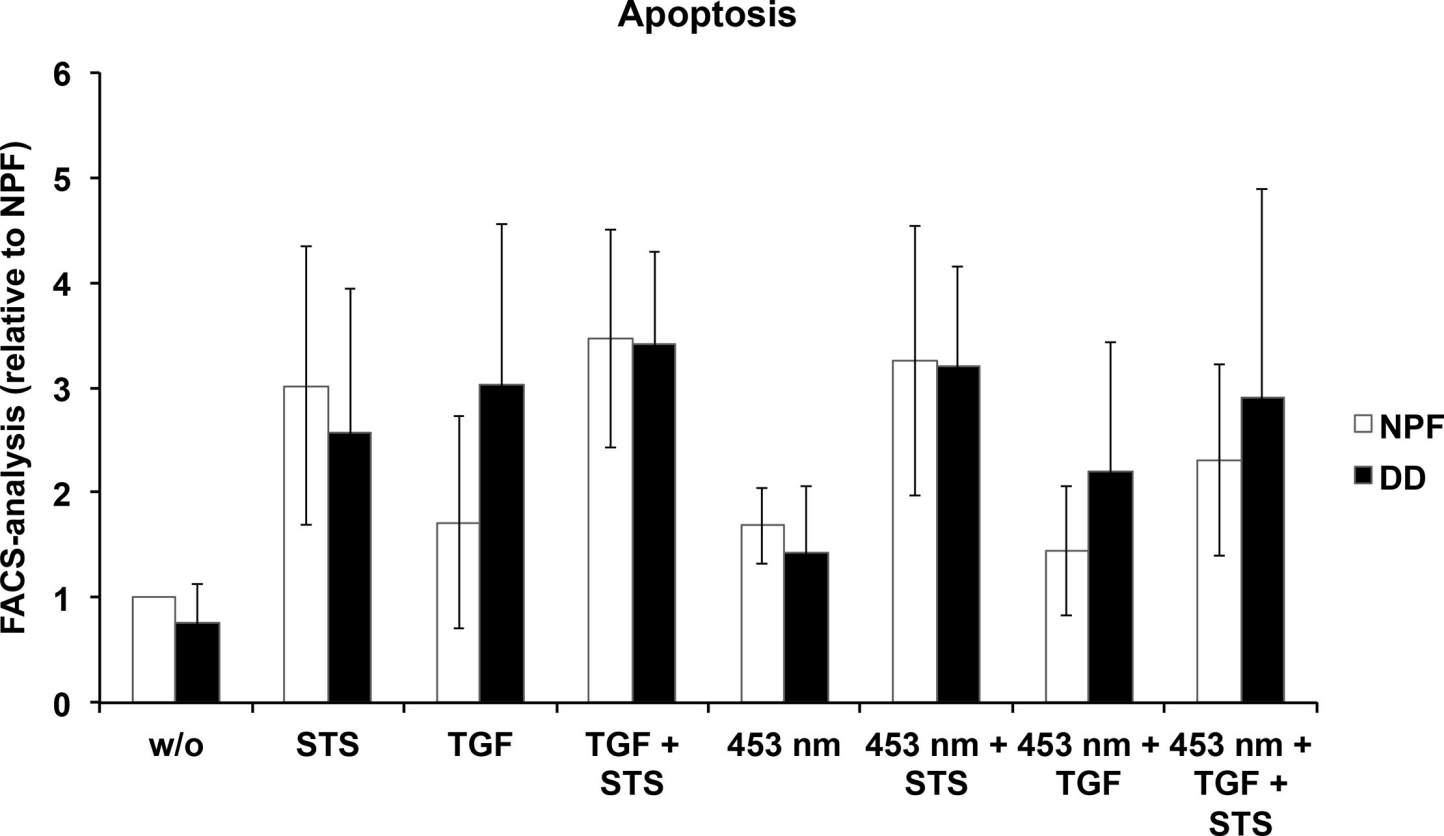
DD fibroblasts reacted with a higher rate of apoptosis compared to NPF fibroblasts. Especially apoptosis of stimulated DD fibroblasts, which were treated with 0.025 and 0.075 μM STS, was significantly enhanced compared to the respective NPF fibroblasts. Relative apoptosis rate of normal palmar fibroblasts (n = 4–7) compared to Dupuytren’s disease fibroblasts (n = 4–6) with or without stimulation of TGF-β1. Cells were treated with 0.025 and 0.075 μm Staurosporin (STS), except the control cells. FACS-Analysis data were normalized to the unstimulated control of NPF fibroblasts. * p ≤ 0.05 bar represent mean ±SD of individual experiments indicated.

The excessive proliferation of myofibroblasts [32], combined with the diminished capability to undergo apoptosis [3, 4] in the proliferative and involutional stages are further characteristics determining the phenotype of DD. In order to proof, if the beneficial effect of blue light is mediated via apoptosis, and to investigate, if photobiomodulation makes Dupuytren’s fibroblasts more sensitive to apoptotic signals, the apoptotic capacity of (Dupuytren’s) fibroblasts was analysed after five days of irradiation and TGF-β1 treatment. To induce cell death staurosporine was used. Interestingly, it was measured that in Dupuytren’s fibroblasts incubated for five days with TGF-β1, apoptosis was significantly higher compared to NPF (Fig 5). But even more important is, that blue light irradiation with a wavelength of λ = 453 nm, intensity of 38 mW/cm^2^, and a radiant exposure of 40 Joule/cm^2^, does not reinforce apoptosis in neither Dupuytren’s fibroblasts nor in NPF fibroblasts.

The present study highlights, that the application of blue light could inhibit the differentiation of Dupuytren's fibroblasts into myofibroblasts and the accompanied α-SMA expression. Our data suggests that, especially in the proliferative and involutional stages, or after surgery when TGF-β concentration is increased, an irradiation with blue light could be beneficial. Consequently, blue light therapy could be a promising therapy option for DD, and especially for relapse prevention. Our results suggest that the usage of blue light is a promising tool for therapeutic treatment for further fibrotic diseases such as keloids, hypertrophic scarring, and scleroderma.
